# A Lecture on Syphilis of the Nervous System

**Published:** 1907-01-19

**Authors:** E. Farquhar Buzzard

**Affiliations:** Assistant Physician to the Royal Free Hospital, the National Hospital for the Paralysed and Epileptic, and the Belgrave Hospital for Children


### Hospital Clinics.

### A LECTURE ON SYPHILIS OF THE NERVOUS SYSTEM.

By E. FARQUHAR BUZZARD, M.A., M.D. Oxon., F.R.C.P. London, Assistant Physician to the Royal Free Hospital, the National Hospital for the Paralysed and Epileptic, and the Belgrave Hospital for Children.

Gentlemen,—I think we shall all agree that syphilis plays a very important rôle in diseases of the nervous system, and that that rôle is not always a direct one. The subject of my lecture to-day is really confined to the direct manifestation of syphilis—the specific lesions—in the nervous system, but I may remind you that syphilis is an important etiological factor in the production of ordinary arteriosclerosis, a disease which is closely associated with cerebral hæmorrhage, cerebral thrombosis, pathologically high arterial tension, and other conditions, all of which give rise to symptoms of nervous origin.

Indirectly, too, syphilis plays a most prominent part, probably through its toxins, in the causation of the so-called "parasyphilitic diseases," of which tabes dorsalis and general paralysis of the insane are the commonest examples. In these morbid processes we do not find specific lesions, but chronic degeneration.

In a third indirect way syphilis is able so to influence the development of the children of syphilitic parents as to lay the foundation of several mental and paralytic diseases. Juvenile tabes and juvenile general paralysis are probably less common results of inherited syphilis than are some less sharply defined forms of mental defect and physical paralysis. On the other hand, there has been in the past a tendency to blame syphilis too much as an ætiological factor. We have no reason to associate it with such common diseases of the nervous system as disseminated sclerosis, progressive muscular atrophy, or amyotrophic lateral sclerosis.

Passing from the indirect to the direct syphilitic lesions of the nervous system, the first point to which I wish to draw your attention is the fact that syphilis exerts its influence practically always and entirely upon the *central* portion of that system. It is true that a peripheral nerve may be accidentally involved in a gummatous growth in any part of the body, but it is doubtful whether we can certainly describe a syphilitic peripheral neuritis. If there is such a condition, comparable to other forms of polyneuritis of toxic origin, it is certainly a very rare disease.

When I say that the central nervous system is the site of specific lesions I mean the whole of it, and do not desire to draw any distinction between the brain and the spinal cord. These may be regarded as one organ in this connection, for the lesions of both are of the same nature and the symptoms evoked only differ in accordance with the difference in function of the parts involved.

The next point is, what are we to call a syphilitic lesion of the nervous system? It has gone out of fashion to draw a sharp line between secondary and tertiary manifestations, and such a distinction is specially inapplicable to the nervous system, because the appearance of identical morbid conditions may be noted within a few months of the primary infection, or may be delayed until a few or many years later. Probably 60 per cent. of syphilitic lesions of the nervous system occur within five years of the chancre. In my experience the cases in which the onset of nervous complications has been exceptionally early have mostly been in young people, but I know of no statistics bearing upon this particular point. Speaking generally, there is no evident association between the severity of the primary sore and the development of the symptoms pointing to gummatous disease of the brain or cord. There are certain general characteristics which apply equally well to cerebral and to spinal syphilis. The most prominent of these is the frequency of multiplicity; often, for example, a gumma of one part is associated with an arterial thrombosis of another. In the same patient we may have evidences of a basal meningitis, and of a cerebral and a spinal thrombosis. The development of each lesion occupies as a rule only a few hours or a few days. A slowly progressive course is rare, except in some cases of syphiloma of the cerebral hemisphere. A third characteristic is the readiness with which syphilitic lesions yield to appropriate treatment, and the readiness also with which they spring up in the same or another situation if neglect of continuity of treatment is displayed either by the doctor or his patient.

We must refer to morbid anotomical facts for the purposes of classification. All syphilitic lesions of the central nervous system arise either in connection with vessels or in connection with the meninges. It is probably true to say that the involvement of cerebral or spinal substance proper is always secondary. Whether the origin is arterial or meningeal the histological process is initially much the same. In either case the first evidence of lesion is a round-celled infiltration of the tissue, which later tends to the formation of fibrous and caseous masses. According as to whether the process is diffuse or local, we may find a diffuse meningitis or a local gumma, or local gummatous arteritis. Probably the latter condition—gummatous arteritis—is to begin with a peri-arteritis—that is to say, an infiltration of the adventitia of the vessel, which interferes with the nutrition of the other coats, with the result that they undergo degenerative changes. This brief description of the anatomy of the syphilitic lesions is sufficient for our purpose to-day, and I will pass at once to the individual forms of the disease as they affect first the brain and secondly the spinal cord.

Cerebral syphilis of any kind is frequently ushered in by prodromal symptoms, the most common of which are headache and insomnia. The headache is often general and ill-defined at first, and later is concentrated in one particular spot which corresponds roughly to the site of the lesion. The insomnia is usually of considerable duration, but is sometimes replaced at or about the time of the onset of definite symptoms by a period of somnolence, at times amounting to stupor.

There are three different kinds of lesions due to cerebral syphilis. The first is vascular, namely, peri-arteritis affecting an artery, of more or less magnitude, which ultimately becomes thrombosed. The symptoms evoked are in no way different from those which we associate with arterial thrombosis due to any other cause, and the exact condition may only be distinguished by general considerations. The latter include such symptoms as headache and somnolence, to which we may add the occasional presence of optic neuritis, which is probably produced by a coincident affection of the basal meninges. The middle cerebral artery is the most frequent site of this vascular disease, and hemiplegia, with or without hemianæsthesia and hemianopia, the most usual clinical manifestation. As a notable example of this condition may be mentioned the case of a man who, at the age of twenty, contracted syphilis, and was treated for a period of seven months. Five months later he developed headache and insomnia, and a few days later, in the course of twenty-four to thirty-six hours, lost the use of the right arm, the right leg, and to a less extent the faculty of speech. He was treated with iodides and mercury energetically, and some improvement rapidly took place, but three weeks later, while still under treatment, he suddenly became drowsy and stupid, and quickly developed a hemiplegia on the left side, became comatose, and died. The autopsy showed typical syphilitic arteritis of both middle cerebral arteries, with thrombotic effects corresponding to the time of their duration. A collateral circulation had been established over a considerable part of the left motor area. The case is characteristic of cerebral vascular syphilis in every respect, except that it did not respond to treatment. These exceptions occasionally occur, and must be regarded as examples of a particularly virulent infection. Basilar arteritis with thrombosis is probably the second most common vascular lesion, and results in pontine thrombosis usually limited to the distribution of one of the branches which leave the main artery at a right angle and pierce the pons along one side of the median raphé. A simple or crossed hemiplegia is thus produced, although in some cases the lesion is more extensive, and a double hemiplegia with pseudo-bulbar symptoms is evolved. I have seen a hemiplegia of pontine origin associated with tabes dorsalis in the same patient, and I may incidentally draw your attention to the fact that syphilitic and parasyphilitic conditions are by no means uncommonly seen together, both clinically and on the post-mortem table.

Thrombosis due to syphilis may involve any cerebral or cerebellar artery, and I have lately observed a case in which the arteries supplying the dentate nuclei of both cerebellar lobes were affected by this condition; but the middle cerebral and basilar vessels are undoubtedly the most common sites for this morbid process.

The next cerebral syphilitic disease is that to which we give the name gumma or syphiloma—a tumour or growth which, like other neoplasms, may be situated either upon or below the surface of the brain substance or upon one of the cranial nerves. Unlike other growths it is probable that gummata always arise in connection with the meninges, although in the cases where they are deeply situated they may extend some distance from the fold of pia-arachnoid whence they take their origin. They are more commonly superficial than deep, and are not infrequently multiple. On account of their situation they frequently give rise to epileptic convulsions, commonly of the Jacksonian type—a convulsion in which the spasm begins in one particular muscle group, spreads with a definite march, and either does or does not become general. In one such case a man had epileptic seizures, beginning in the hand and unaccompanied by loss of consciousness, for five years before he came under observation. The development of further syphilitic lesions proved fatal, and a superficial gumma in the hand area of the ascending frontal convolution was found at the autopsy.

Gummatous meningitis or gummatous meningo-encephilitis is the third type of cerebral syphilis. Its favourite site is probably that part of the basal meninges which corresponds to the interpeduncular space, where the basilar artery breaks up to take its part in the formation of the circle of Willis, and where the third nerves lie in close contact with the posterior cerebral arteries. As a result of this anatomical arrangement, meningitis in that region is most frequently manifested clinically by a unilateral or bilateral ophthalmoplegia, not uncommonly associated with headache, vomiting, and optic neuritis. This clinical picture as the first evidence of intracranial syphilis is by no means unusual.

Another frequent site for the meningeal gummatous process is the posterior end of the fourth ventricle. This condition is a most serious one, because, in spite of the fact that it may be recognised early and appropriately treated, it nearly always gives rise to hydrocephalus by blocking the exit of the cerebro-spinal fluid. In fact, the fibrous healing which takes place as the acute stage subsides is probably largely instrumental in bringing about the blockage. Those cases which occur in children are easily recognised on account of the progressive enlargement of the skull, but in the adult the clinical picture is often that of an intracranial tumour without localising signs. Such a case came under my observation last year, and led me to advise trephining the right frontal region for the relief of pressure and for the possible presence of a tumour in that situation. No tumour was found, but for some months the patient was free from symptoms. These, however, returned, and in spite of anti-syphilitic treatment the man died. A gummatous meningitis was found in a healed condition at the posterior end of the fourth ventricle. In these cases we cannot expect to get good results from the exhibition of mercury and iodides, and so far surgery is inadequate to deal with the difficulty.

Less commonly the surface of the hemisphere is attacked by a meningitis which eventually leads to involvement of the dura mater as well as the leptomeninges. A thick fibrous mass becomes formed upon the cortical surface, and the latter frequently undergoes secondary necrosis. This condition is known as pachymeningitis, and the symptoms it gives rise to are often rather obscure. Epileptic convulsions are common, and I have seen a victim of this morbid process die in *statu epileptico*. Here again the lesion, once established, is practically beyond remedial treatment.

I now pass to the spinal cord. The prodromal symptoms are usually defined as tingling or numbness in various parts of the limbs or trunk, not infrequently associated with pain of a burning character located over some part of the vertebral column.

Again, the first group of cases may be classed as vascular. Many years ago Dr. Bastian pointed out that spinal thrombosis was a frequent cause of the condition improperly known as transverse myelitis. True, inflammatory myelitis is a comparatively rare disease, and statistics show that 80 per cent. of cases to which the term myelitis has been applied are probably cases of spinal thrombosis, the majority of syphilitic origin. The anatomical process is identical with that seen in cerebral thrombosis, and the resulting necrosis of tissue gives rise to symptoms of paralysis and anæsthesia corresponding to the position of the lesion. Like cerebral thrombosis, too, spinal thrombosis has a favourite site—*i.e.* in the dorsal region of the cord between the sixth and eleventh dorsal segments. Most frequently, therefore, the clinical picture of such a case is that of a paraplegia which may be either flaccid or spastic. When the lesion amounts to a complete physiological section of the cord, a flaccid paralysis of the lower limbs is produced; when the lesion is incomplete, a spastic paraplegia is the result. Other phenomena in relation to sensation, to the action of the sphincters, and to trophic changes in the muscles, depend upon the extent and site of the disease.

Gummata are found in the substance of the cord and sometimes on the spinal roots. They are not infrequently multiple. When they occur in the substance of the cord they produce the symptoms which we associate with other kinds of intramedullary neoplasm, and they sometimes simulate the picture of a transverse myelitis. Spastic paraplegia in a syphilitic patient may be due either to a gumma or to a focal thrombosis. Fortunately the treatment is on the same lines in either case.

With regard to the spinal meninges, we recognise here too a leptomeningitis and a pachymeningitis, the former being more common on the posterior surface of the dorsal region, the latter—a rare affection—involving, as a rule, the cervical enlargement. The symptoms are often somewhat indefinite, but in the main belong to the following type; atrophic paralysis of muscular groups of similar segmental innervation, and pain, together with impairment of sensation in the area of distribution of the spinal nerves, are most often observed.

In regard to the general diagnosis of these diseases the facts I have already put before you, especially those which I have referred to as characteristic of the cerebral and spinal types, are of great importance, and, together with the necessary inquiry into the antecedents of the patient, the observation of evidence of syphilitic lesions in other systems, and the results of treatment, are usually sufficient for our needs. By means of lumbar puncture an examination of the cerebro-spinal fluid for excess of lymphocytes may in a very few cases be of service, when other signs are wanting, but this procedure can hardly be regarded as a *sine quâ non* for the purpose of diagnosis, and is certainly not yet justified as a routine measure in all cases. It is important not to be misled by the tendency to partial recovery or improvement which is evinced by almost all cases of intracranial neoplasm when placed in bed and under the influence of mercury and iodides. This is probably merely the effect of lowered blood-pressure. On the other hand, the presence of the Argyll Robertson pupil in association with lesions which are obviously of a gross character, is a piece of evidence in favour of their syphilitic origin not to be overlooked.

*Treatment.*—Everyone has his own way of administering mercury. Probably the application of mercury by inunction and the giving of iodide by the mouth is as good as any. The important thing is to push the former to the point of toleration, and to give sufficient doses of iodide for the purpose in view. It is not necessary to give more than thirty or forty grains three times daily in the majority of cases, and this dosage should be reached by gradual additions. Above all it is necessary to remember that a patient who has once manifested a syphilitic process in his nervous system should be as continually under medical care as an epileptic, especially when he is feeling most fit and well. The judicious administration of mercury at regular intervals over many years, preferably for the remainder of life, is strongly indicated in every case of cerebro-spinal syphilis, and I am firmly of the opinion that by this means, and by this means only, can we ward off a recurrence of the disease. As I

have already pointed out, some very exceptional cases are not responsive even to energetic treatment.

It is not often that cases of cerebral syphilis require surgical treatment, but the free opening of the skull for the sake of rapidly ameliorating the optic neuritis when this has gone very far and permanent loss of sight is threatening, and when anti-syphilitic treatment has been too long delayed, may certainly be considered justifiable in exceptional cases.

